# Bio-based reactive diluents as sustainable replacements for styrene in MAESO resin†

**DOI:** 10.1039/c8ra00339d

**Published:** 2018-04-12

**Authors:** Yuehong Zhang, Yuzhan Li, Vijay Kumar Thakur, Liwei Wang, Jiyou Gu, Zhenhua Gao, Bo Fan, Qiong Wu, Michael R. Kessler

**Affiliations:** College of Material Science and Engineering, Northeast Forestry University Harbin 150040 P. R. China; School of Mechanical and Materials Engineering, Washington State University Pullman WA 99164 USA Michael.R.Kessler@ndsu.edu +1-701-231-7494; College of Chemical Engineering, Qingdao University of Science and Technology Qingdao Shandong province 266042 P. R. China; Enhanced Composites and Structures Center, School of Aerospace, Transport and Manufacturing, Cranfield University Bedfordshire MK43 0AL UK; Department of Mechanical Engineering, North Dakota State University Fargo ND 58108 USA

## Abstract

Four different biorenewable methacrylated/acrylated monomers, namely, methacrylated fatty acid (MFA), methacrylated eugenol (ME), isobornyl methacrylate (IM), and isobornyl acrylate (IA) were employed as reactive diluents (RDs) to replace styrene (St) in a maleinated acrylated epoxidized soybean oil (MAESO) resin to produce bio-based thermosetting resins using free radical polymerization. The curing kinetics, gelation times, double bond conversions, thermal–mechanical properties, and thermal stabilities of MAESO-RD resin systems were characterized using DSC, rheometer, FT-IR, DMA, and TGA. The results indicate that all four RD monomers possess high bio-based carbon content (BBC) ranging from 63.2 to 76.9% and low volatilities (less than 7 wt% loss after being held isothermally at 30 °C for 5 h). Moreover, the viscosity of the MAESO-RD systems can be tailored to acceptable levels to fit the requirements for liquid molding techniques. Because of the introduction of RDs to the MAESO resin, the reaction mixtures showed an improved reactivity and an accelerated reaction rate. FT-IR results showed that almost all the C=C double bonds within MAESO-RD systems were converted. The glass transition temperatures (*T*_g_) of the MAESO-RDs ranged from 44.8 to 100.8 °C, thus extending the range of application. More importantly, the *T*_g_ of MAESO-ME resin (98.1 °C) was comparable to that of MAESO-St resin (100.8 °C). Overall, this work provided four potential RDs candidates to completely replace styrene in the MAESO resin, with the ME monomer being the most promising one.

## Abstract 2

This paper reports four promising, sustainable reactive diluents to completely replace styrene for a commercially available MAESO resin.

## Graphical abstract

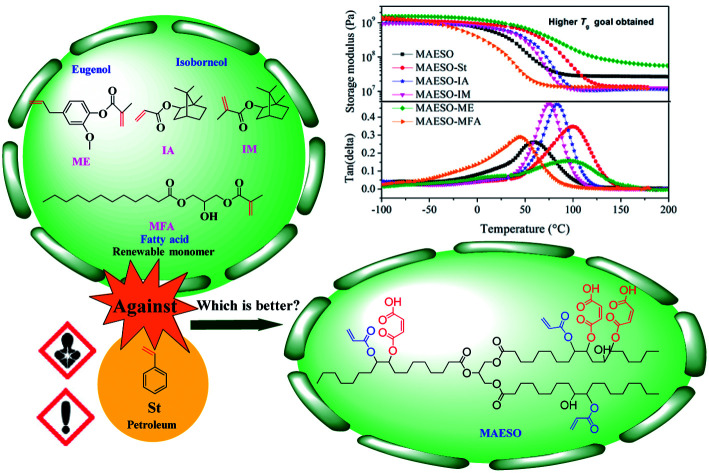

## 1. Introduction

High performance thermosetting resins, such as vinyl ester resins,^1,2^ unsaturated polyester resins,^3^ and epoxy resins,^4,5^ to name a few, are widely used as matrices for fiber reinforced composites, owing to their outstanding thermal–mechanical properties, easy processability, light weight, and relatively low cost. However, these resins are non-degradable, difficult to recycle, and derived from nonrenewable fossil fuels, leading to serious challenge after their service life by putting heavy burden on the environment. Driven by the depletion of nonrenewable fossil fuels, stringent governmental regulations, together with increasing environmental concerns, significant research efforts have been triggered to develop bio-based materials from renewable resources in both academia and industry.^6^

The most widely used renewable resources include vegetable oils, polysaccharides (mainly cellulose, and starch), lignin, natural rubber, and proteins.^7–9^ Among them, soybean oils are one of the most widely investigated platform chemicals to replace petroleum-based chemical in the preparation of bio-based thermosets because of their abundant availability, sustainability, and relatively low cost.^10–12^ Soybean oils are composed of approximately 99% of triglycerides. Soybean oils contain mainly five fatty acids: palmitic acid (10%), stearic acid (4%), oleic acid (18%), linolenic acid (13%), and linoleic acid (55%). The presence of non-conjugated C=C double bonds within these fatty acids are not sufficiently reactive to be polymerized directly.^13–16^ Therefore, a series of polymerizable functional groups, such as epoxy, methacrylate, and acrylate groups may be introduced to convert soybean oil into thermosetting resins. Acrylated epoxidized soybean oil (AESO) and maleinated acrylated epoxidized soybean oil (MAESO) have been widely reported to replace petroleum-based vinyl ester resin or unsaturated polyester resin.^17–19^ AESO is obtained *via* epoxidation of soybean oil followed by acrylation. MAESO is formed by further maleination of AESO (ESI Fig. S1†). Thus, MAESO resin possesses more reactive C=C double bonds than that of AESO resin.^20,21^ Generally, pure MAESO resin is too viscous to use, and therefore demands approximately 35% of styrene as a reactive diluent before being cured by a free radical polymerization. However, new emission standards for composite manufacturing by the Environmental Protection Agency specifically have targeted styrene as a regulated hazardous air pollutant (HAP) and volatile organic compound (VOC).^22^ Styrene is also reported as a potential human carcinogen and is derived from non-renewable petroleum. Thus, reducing the emissions from polymer composite formulations is critical to comply with environmental regulations. Therefore, developing biobased, sustainable, green RDs alternatives to styrene are receiving great interest.

In recent years, a series of bio-based vinyl monomers, mainly acrylates and methacrylates, have been developed to replace styrene as reactive diluents for vinyl ester resins and unsaturated polyester resins,^23^ such as methacrylated fatty acid (methacrylated lauric acid, methacrylated hexanol, and methacrylated oatanoil acid),^24–27^ methacrylated isosorbide (from isosorbide, a low cost heterocyclic compound derived from glucose),^28,29^ methacrylated cardanol (from cashew nut shell liquid, a natural phenol),^30^ methacrylated lignin model derivatives (vanillin, guaiacol, syringols, and catechols),^31–33^ acrylated sucrose,^34^ furfuryl methacrylate (from furfural, an abundant organic compound derived from a variety of agricultural byproducts),^35^ and methacrylated rosin derivatives.^36^ However, these reported bio-based reactive diluents have some drawbacks: (1) the availability of some renewable resources is not industrially mass-produced. (2) These RDs usually showed a significantly higher viscosity than that of styrene. The increased viscosity negatively affects the processability of matrix resin and makes it difficult to ensure good wetting of fibers. (3) The use of these RDs resulted in thermosets with low modulus, mechanical strength, and *T*_g_, which does not meet industrial needs.^37^

**Fig. 1:**
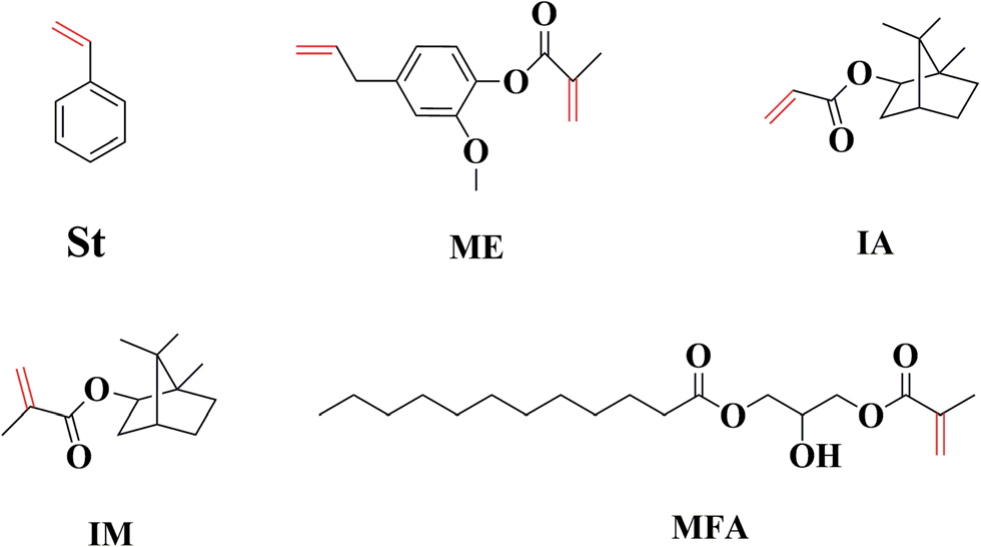
Chemical structure of five RDs.

Therefore, efficient and economic RDs are required to further facilitate the commercial applications of MAESO resin. We propose to evaluate four bio-based monomers (IA, IM, ME, and MFA in Fig. 1) as potential RDs. All of them possess unsaturated C=C double bonds similar to St, and can be derived from inexpensive and abundant biorenewable building blocks: IM and IA (from isoborneol, prepared from reduction of camphor), ME (from eugenol, typically extracted from clove, and pyrolysis or depolymerization of lignin), and MFA (from fatty acid, prepared from vegetable oil). Moreover, IM, IA, and ME can be prepared by esterification using the corresponding alcohol with methacrylic/acrylic acid or methacrylic/acrylic anhydride without using any solvent,^31^ while MFA can be prepared by reacting different types of fatty acid with glycidyl methacrylate.^24^

In the present work, four bio-based methacrylate/acrylate candidates (IM, IA, ME and MFA) were evaluated as potential RDs to replace styrene in a commercialized MAESO resin. The viscosities and the volatilities of the RDs were analyzed and compared to styrene. Furthermore, the curing kinetics, gelation times, double bond conversions, thermal–mechanical properties, and thermal stabilities of the MAESO-RDs resin were evaluated and compared to the benchmark styrene systems.

## 2. Experimental section

### 2.1 Materials

Eugenol (98%), styrene, methacrylic anhydride (94%, inhibited with 2000 ppm topanol A), 4-dimethylaminopyridine (DMAP), and *tert*-butyl peroxybenzoate were provided by Sigma-Aldrich. Dichloromethane, sodium bicarbonate, sodium hydroxide, and anhydrous magnesium sulfate were provided by Fisher Scientific. Hydrochloric acid was provided by EMD Millipore. MAESO (yellow to amber viscous liquid, 1.02 g cm^−3^ at 25 °C, with approx. 15% of maleic anhydride in MAESO, the level of maleic functionality was 1.45, and the acrylate group was 3.16) and MFA (MC818) were supplied by Dixie Chemical Company, Inc. IM and IA were obtained from Ark Pharm, Inc. All chemicals were used as received without further purification.

### 2.2 Synthesis of ME

**Fig. 2:**
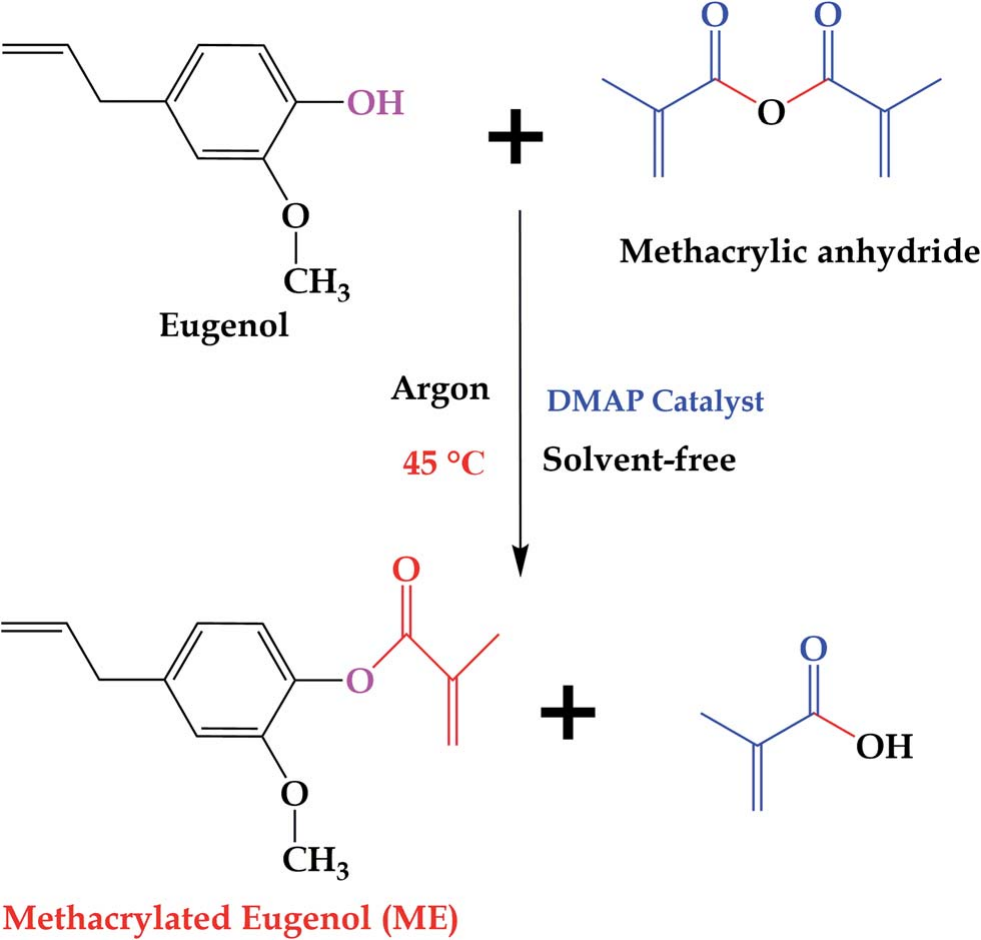
Synthesis of ME.

10 g of eugenol and 0.157 g (2% mole equivalents based on methacrylic anhydride) of DMAP were introduced to a 100 mL flask, and the system was sealed and purged with argon for 2 h. After that, 10.33 g (1.1 mole equivalents based on eugenol) of methacrylic anhydride was added. The final mixture was heated at 45 °C under stirring for 24 h, and then allowed to wash with saturated sodium bicarbonate aqueous solution several times, and extracted with dichloromethane to remove the unreacted methacrylic anhydride and methacrylic acid. The obtained organic layer was sequentially treated with 1.0 M NaOH aqueous solution, 0.5 M NaOH aqueous solution, 1.0 M HCl aqueous solution, and water. Finally, a pale yellow product was obtained after drying over MgSO_4_, filtering, concentrating under reduced pressure, and drying in a vacuum oven at 60 °C overnight. Fig. 2 shows the synthesis route of ME.

### 2.3 Preparation of MAESO-RD thermosetting resins

MAESO was heated to 70 °C for 10 min to decrease the viscosity, and then it was blended with five different kinds of RDs (ME, IM, IA, St and MFA), respectively, while maintaining the weight ratios of MAESO to RD at 65 : 35. Then 1.5 wt% of *tert*-butyl peroxybenzoate was introduced as free radical initiator. The mixture was poured into a preheated steel mold and was transferred to a vacuum oven to remove air bubbles. The resins were cured at 90 °C for 1 h, 130 °C for 5 h, and were post-cured 180 °C for 2 h. In order to prevent oxygen free radical inhibition, the mixture was purged with argon prior to the curing. The prepared thermosets were labeled as pure MAESO, MAESO-ME, MAESO-IM, MAESO-IA, MAESO-St, and MAESO-MFA.

### 2.4 Resin characterization

Proton nuclear magnetic resonance (^1^H NMR) spectra was recorded on a Varian VXR-300 NMR instrument at 25 °C using DMSO-d6 as a solvent.

The volatility behavior was studied using a thermogravimetric analyzer (TGA) (Discovery TGA, TA Instrument). Samples were isothermally heated at 30 °C for 5 h under a nitrogen flow of 25 mL min^−1^.

The viscosity was measured using a strain-controlled rheometer (ARES-G2, TA Instruments). Measurements were performed isothermally using a parallel-plate geometry (25 mm diameter) with shear rates ranging from 1 to 100 s^−1^ at 30, 35, 40, 45, 50, 55, and 60 °C, respectively.

The curing kinetics of MAESO-RDs were investigated using a differential scanning calorimeter (Discovery DSC, TA Instruments) in a dynamic mode at various heating rate: 5, 10, 15, and 20 °C min^−1^ from room temperature to 280 °C. The total reaction heat (Δ*H*) of the curing reaction was obtained from the integrated area under the exothermic peak. Activation energy of the curing reaction was determined from the peak temperature at different heating rates using the following Kissinger's theory,^29^ln(*β*/*T*_p_^2^) = ln(*AR*)/*E*_a_ − *E*_a_/*RT*_p_where *β* is the heating rate, *T*_p_ is the exothermic peak temperature, *A* is the pre-exponential factor, *E*_a_ is the activation energy, and *R* is the gas constant.

The gelation time of the resins was measured on the ARES-G2 rheometer using 25 mm diameter parallel plates with time sweeps at a constant shear frequency of 1.0 Hz at 100 °C.

The collection of FT-IR were performed using a NEXUS 670 FTIR spectrometer in attenuated total reflectance mode.

Dynamic mechanical analysis (DMA) was carried out on the ARES-G2 rheometer in DMA mode with a torsion geometry. Samples were heated at 3 °C min^−1^ from −100 to 200 °C, with a strain of 0.065%, and a constant frequency of 1 Hz.

Thermogravimetric behavior of the resins was investigated using a Discovery TGA from TA Instruments. All samples were tested from room temperature to 600 °C at a heating rate of 10 °C min^−1^ under a nitrogen flow of 25 mL min^−1^.

## 3. Results and discussion

**Fig. 3:**
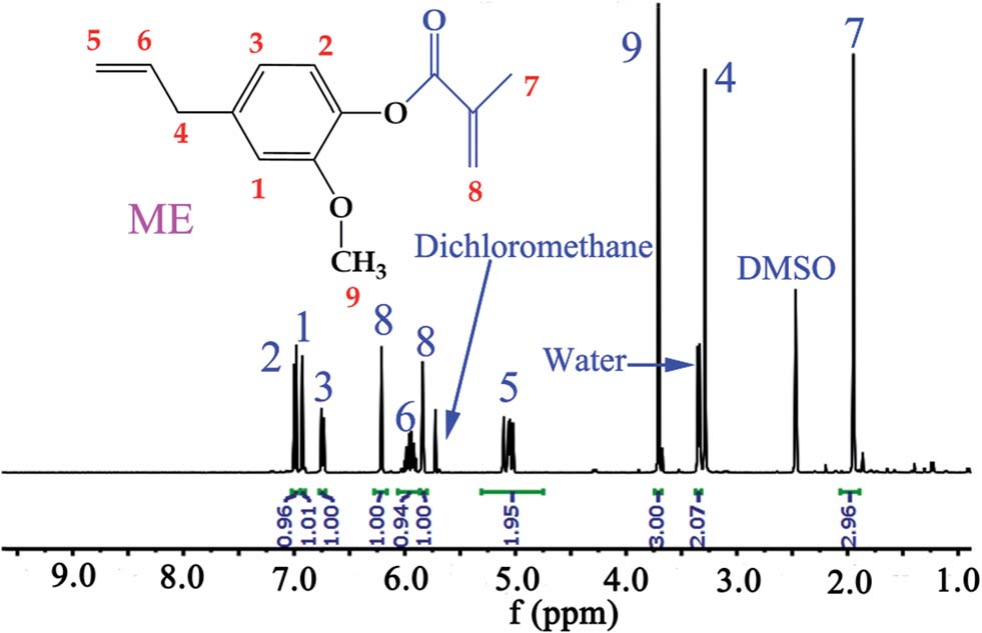
^1^H NMR spectrum of ME monomer.

In this work, ME was synthesized according to reported procedures,^14,33^ while MFA, IM, and IA are all commercially available products. Specifically, eugenol was functionalized with reactive methacrylate group, using methacrylate anhydride, in the presence of DMAP catalyst, under mild conditions by one pot Steglich esterification reaction to obtain ME monomer, and the average yields was 81.8% after aqueous washing. The chemical structure of the synthesized ME was confirmed by ^1^H NMR spectrum (Fig. 3), the chemical shift *δ* (ppm) was observed as follow: 6.99, 6.92, 6.74 (2.94H, Ar–H), 6.21, 5.84 (2H, –C=CH_2_), 5.95 (1H, =CH–), 5.06 (1.95H, CH_2_=), 3.71 (3H, –OCH_3_), 3.34 (2.07H, –CH_2_–), 1.95 (2.96H, –CH_3_).

**Fig. 4:**
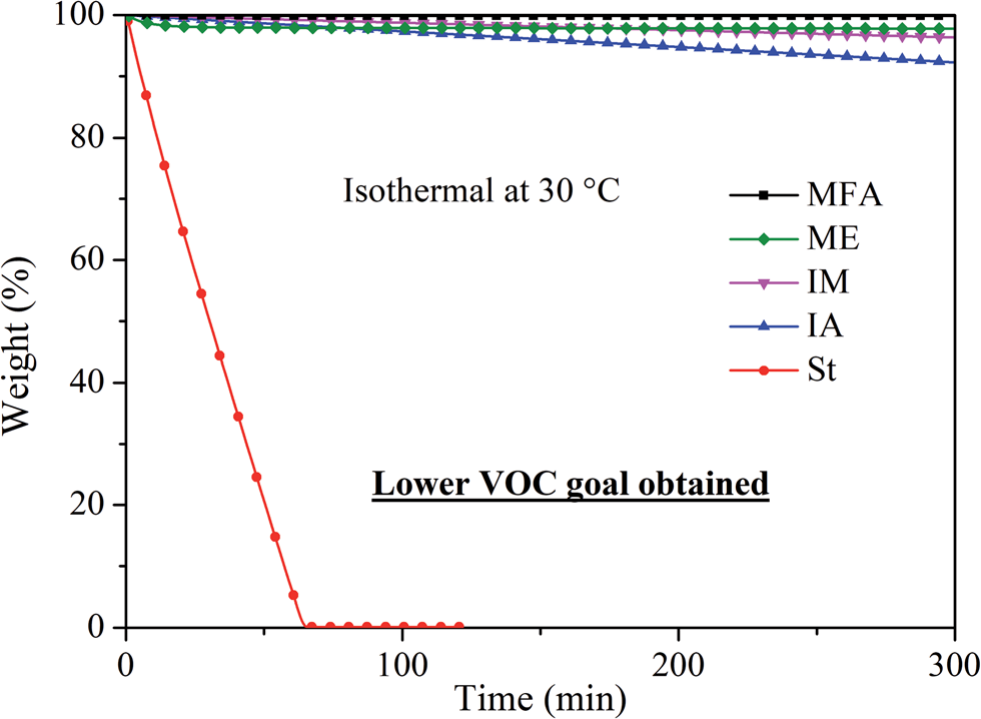
Volatilities of five RDs.

Volatility is an important factor to consider when designing novel RDs because volatile RDs are more likely to be emitted even after incorporated into polymer composites, leading to potential health hazards. As shown in Fig. 4, styrene completely evaporated within 65 min at 30 °C. In contrast, IA, IM, MFA, and ME monomer exhibited significantly low volatility with a weight loss of 7.8%, 3.7%, 0.2%, and 2.2%, respectively, after being held isothermally at 30 °C for 5 h, indicating they are all promising candidates for RDs.

In addition, the sustainability of the five different RDs monomers and MAESO resin was quantified using the biobased carbon content (BBC) and is summarized in Table 1. It is worth noting that the BBC of St is 0, while the BBC of IA, IM, MFA, and ME range from 63.2 to 76.9% because they are all derived from bio-based precursors such as eugenol, fatty acid, and isoborneol. These methacrylated/acrylated monomers are generally synthesized through the reaction between bio-based precursor and methacrylic acid or methacrylic anhydride. It is estimated that methacrylic acid has great potential to be produced from renewable resources, such as fermentation of sugars, at industrial scale by 2018,^38^ and thus these four RDs will have the BBC of 100% in the future. In addition, the BBC of MAESO resin is 73.1%, further improving sustainability of the resulting MAESO-RD resins.

**Table 1:** The BBC of MAESO and RD monomers

Formulations	Bio-based precursors	Bio-based carbon content (%)
MAESO	Soybean oil	73.1
MFA	Fatty acid	63.2
ME	Eugenol	71.4
IM	Isoborneol	71.4
IA	Isoborneol	76.9
St	—	0

**Fig. 5:**
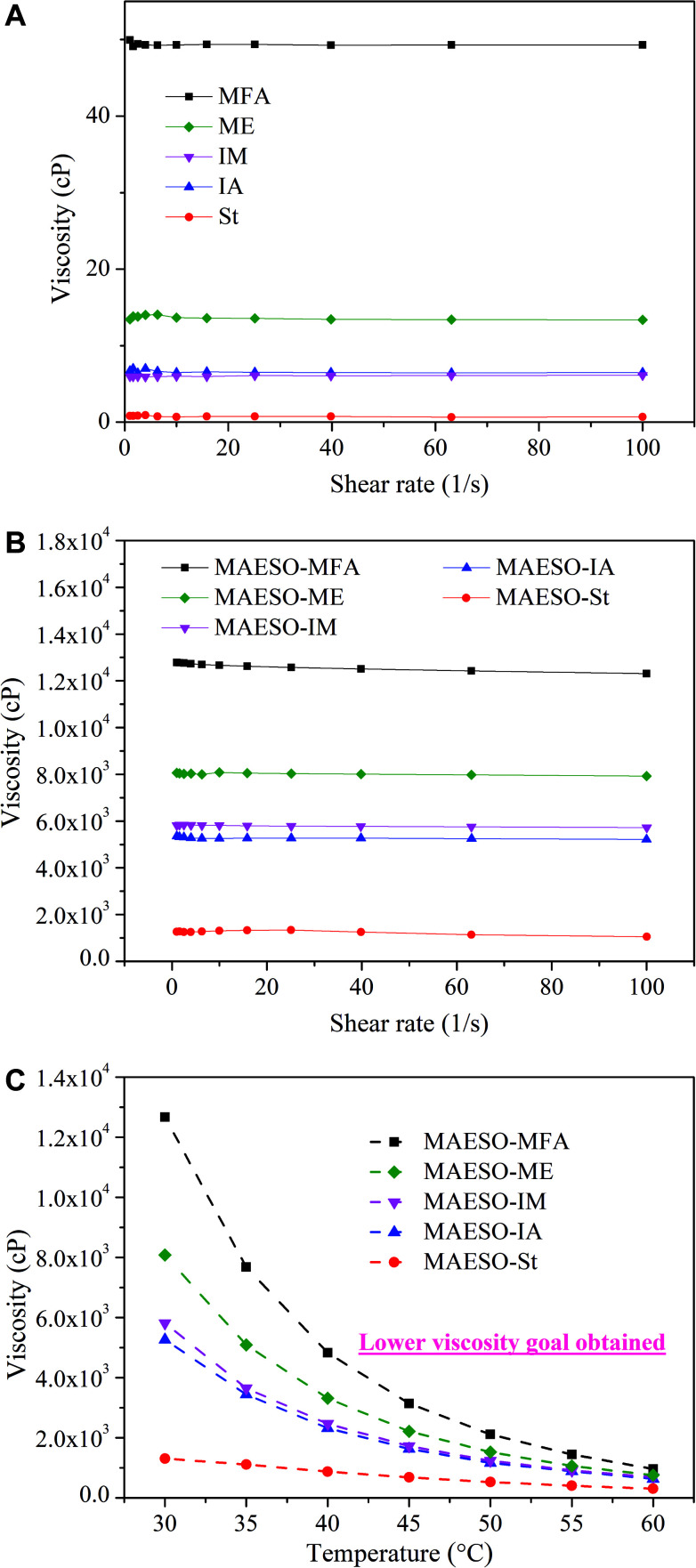
(a) Viscosities of five RDs monomers at 30 °C. (b) Viscosities of MAESO-RD systems at 30 °C. (c) Viscosities of MAESO-RD system as a function of temperature at shear rate of 10 s^−1^.

Generally, reactive diluents play two roles in the preparation of MAESO-RD resins. First, they serve as solvents to reduce the viscosity of MAESO resin so that it can be processed using various industrial techniques. Therefore, low viscosity is favorable for RD monomers. The viscosities of IA, IM, MFA, and ME are 6.5, 6.0, 49.3, and 13.7 cP with a shear rate of 10 s^−1^ at 30 °C (Fig. 5(a)), respectively, which are all higher than that of St (0.7 cP), because St is a non-polar, low molecular weight molecule. Pure MAESO resin has an extremely high viscosity of 7.5 × 10^5^ cP at 30 °C.^14^ This is because of its high molecular weight, and abundance of hydrogen bonds, which greatly increase the dissipation of internal energy. By blending with RD monomers, the resins viscosities were greatly decreased and followed the order of *η*_MAESO-MFA_ > *η*_MAESO-ME_ > *η*_MAESO-IM_ > *η*_MAESO-IA_ > *η*_MAESO-St_ (Fig. 5(b)). This was mainly attributed to the low viscosity of the pure RD monomers, and the MAESO-St system showed the lowest viscosity (1300 cP at 30 °C).

Moreover, the viscosities of the MAESO-RD systems can be further reduced by increasing processing temperature (Fig. 5(c)), which was supported by the Arrhenius equation:

1 *η* = *η*_0_ exp(*E*_*η*_/(*RT*))

where *η* is the apparent viscosity, *η*_0_ is the prefactor, *E*_*η*_ is the activation energy for the viscous flow, *R* is the ideal gas constant, and *T* is the absolute temperature. As shown in Table 2, *E*_*η*_ values exhibited the same trend as the viscosity values of the MAESO-RD system (Fig. 5(b)). The MAESO resin showed the highest *E*_*η*_ of 101.6 kJ mol^−1^, while the MAESO-RD systems exhibited reduced *E*_*η*_ values ranging from 71.5 kJ mol^−1^ for MAESO-MFA to 58.5 kJ mol^−1^ for MAESO-IA. This was attributed to the introduction of RDs into MAESO resin, which increased flowability and thus greatly decreased the *E*_*η*_.

**Table 2:** Viscosities of MAESO-RD system at 30 °C and the calculated Arrhenius parameters

Formulations	*η* (Pa s, 30 °C)	*η*_0_ (Pa s)	*R*_2_	*E*_*η*_ (kJ mol^−1^)
MAESO	759.0	2.48 × 10^−15^	0.9967	101.6
MAESO-MFA	12.7	5.95 × 10^−12^	0.9994	71.5
MAESO-ME	8.1	3.15 × 10^−11^	0.9993	66.1
MAESO-IM	5.8	3.35 × 10^−10^	0.9968	59.3
MAESO-IA	5.3	4.13 × 10^−10^	0.9982	58.5
MAESO-St	1.3	1.23 × 10^−7^	0.9921	41.0

The optimal viscosity range for liquid molding resins is below 1000 cP. Based on the relationship between viscosity and temperature, by increasing the processing temperature of the MAESO-RD systems to 60 °C, the viscosities of MAESO-MFA, MAESO-ME, MAESO-IM, and MAESO-IA can be reduced to 961, 633, 689, 756 cP, respectively, which can match the viscosity requirement for liquid molding techniques.

The second role of RD is to copolymerize with the C=C double bonds of MAESO resin to improve the overall properties of the network polymer. Dynamic DSC scans (ESI Fig. S2–S6†) were used to determine the polymerization behavior of the MAESO-RD systems in the presence of 1.5% *tert*-butyl peroxybenzoate as a free radical initiator. Both RDs (methacrylate/acrylate/allylic groups) and MAESO (acrylate group and maleate group) resins can undergo free radical polymerization at elevated temperature, resulting in an exothermic peak.

*tert*-Butyl-peroxybenzoate has been commonly employed as an initiator in free radical polymerization because of its thermally-sensitive peroxide structure, which decomposes in the temperature range of 105–205 °C and reaches the fastest decomposition rate at 165 °C. Therefore, all the MAESO-RD systems exhibited exothermic peaks between 100 and 250 °C (ESI Tables S1–S5†).

For pure MAESO resin with *tert*-butyl-peroxybenzoate, two exothermic peaks were observed, see Table 3, corresponding to the free radical polymerization of double bonds. The first peak (120–200 °C) showed an exothermic enthalpy of 157.07 J g^−1^, while the second one (200–250 °C) had a significantly lower enthalpy of 9.24 J g^−1^. This was attributed to the incomplete polymerization of MAESO resin under 200 °C. With further increasing the temperature to 250 °C, some unreacted double bonds of MAESO and more free radicals from *tert*-butyl-peroxybenzoate were released, resulting in a second exothermic peak.

**Table 3:** Curing kinetics of MAESO-RD systems

Monomers	Peaks	5 °C	10 °C	15 °C	20 °C	Exothermal enthalpy (J g^−1^)	Activation energy (*E*, kJ mol^−1^)	*R* ^2^
MAESO	P1	149.35	156.97	160.53	162.86	157.07	148.11	0.993
	P2	201.59	211.51	217.92	221.71	9.24	126.01	0.999
MAESO-ME	P1	136.70	146.00	150.64	154.23	239.20	108.17	0.998
MAESO-MFA	P1	126.75	133.29	136.52	139.05	186.83	130.99	0.995
	P2	193.28	204.86	211.28	215.89	2.18	108.12	0.999
MAESO-IA	P1	138.66	145.79	150.09	152.24	228.56	138.83	0.996
	P2	199.57	209.02	215.60	220.53	3.93	120.44	0.998
MAESO-IM	P1	125.84	131.89	136.00	138.73	211.25	139.62	0.999
	P2	198.67	208.94	216.04	220.11	4.08	115.64	0.999

As shown in Table 3, the activation energy involved in the first exothermic peak for MAESO resin was the highest with a value of 148.11 kJ mol^−1^ due to its low reactivity. With the introduction of RDs, the activation energy values for MAESO-RD resins were greatly decreased, and the exothermic enthalpy was significantly higher, indicating that the introduction of RDs accelerated the free radical polymerization rate and facilitated the conversion of C=C double bonds within the resin. This can be explained by two reasons: (1) the introduction of RDs greatly decreased the viscosity of MAESO-RDs systems, thus facilitating the polymerization, and (2) the introduction of RDs complicated the curing process of the MAESO-RD systems, which involved MAESO homopolymerization, RD homopolymerization, and MAESO-RD copolymerization. The activation energy values for MAESO-RD systems were in the order of *E*_MAESO_ > *E*_MAESO-IM_ > *E*_MAESO-IA_ > *E*_MAESO-MFA_ > *E*_MAESO-ME_. The activation energy for the MAESO-ME system showed the lowest value of 108.17 kJ mol^−1^, which was 40 kJ mol^−1^ lower than that of pure MAESO resin, indicating highest reactivity of the MAESO-ME system. We attribute this to the fact that ME monomer has more C=C double bonds (methacrylate groups and allylic groups) per gram that can participate in the polymerization this was also confirmed by the higher exothermic enthalpy of the MAESO-ME system than that of pure MAESO resin. Generally, higher exothermic enthalpy of MAESO-RD systems indicates high conversion of C=C double bonds. The exothermic enthalpy values for MAESO-RD systems were in the order of Δ*H*_MAESO_ < Δ*H*_MAESO-MFA_ < Δ*H*_MAESO-IM_ < Δ*H*_MAESO-IA_ < Δ*H*_MAESO-ME_, which is in accordance with their C=C double bonds concentration since the molecular weight of RDs was in the order: *M*_IA_ < *M*_IM_ < *M*_ME_ < *M*_MFA_ (ME has two double bonds per molecular).

**Fig. 6:**
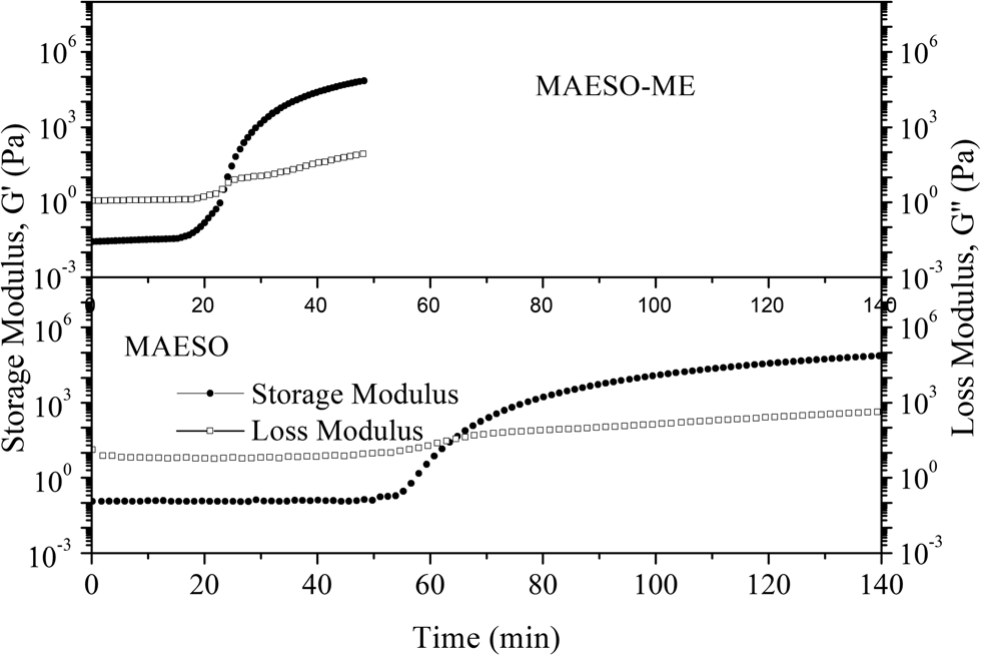
Time dependence of *G*′ and *G*′′ for MAESO-RD resins.

The curing behavior of the MAESO-RD systems was further investigated using rheological tests, in which the storage modulus (*G*′) and loss modulus (*G*′′) were monitored throughout the isothermal curing process of the MAESO-RD resins at 100 °C. The time at which *G*′ crosses over *G*′′ is defined as the gelation time. As shown in Fig. 6, prior to curing, *G*′′ was significantly higher than *G*′ indicating a liquid-like viscous behavior. When isothermally cured at 100 °C, *G*′ increased faster than *G*′′, which was attributed to the free radical polymerization and indicated that solid-like behavior dominated the system. The gelation time of pure MAESO resin was 63.6 min (Table 4). After introducing RDs, the gelation time was significantly decreased. The MAESO-St resin system exhibited the lowest gelation time of 15.1 min, and the gelation time of MAESO-RDs was in the order of *t*_MAESO_ > *t*_MAESO-IM_ > *t*_MAESO-IA_ > *t*_MAESO-MFA_ > *t*_MAESO-ME_ > *t*_MAESO-St_. This was attributed to the different reactivities between MAESO and RDs, as confirmed by the activation energy value in Table 3.

**Table 4:** Gelation time for MAESO-RD resins

Formulations	Gel time (*t*, min)
MAESO	63.6
MAESO-MFA	25.2
MAESO-ME	23.4
MAESO-IM	35.8
MAESO-IA	30.5
MAESO-St	15.1

The free-radical-initiated reaction in the MAESO-RD systems was highly complicated, which involved MAESO homopolymerization, RD monomer homopolymerization, and MAESO-RD copolymerization. Though maleic groups (MAESO) do not readily homopolymerize, it can not only copolymerize with meth(acrylate) groups (RD), but also copolymerize with acrylate groups (MAESO). In addition, the high viscosity and the long chain entanglement also have great effect on the curing extent. Therefore, it's hard to analysis and quantifies the reactivity ratio between the MAESO and acrylate or methacrylate monomers.

**Fig. 7:**
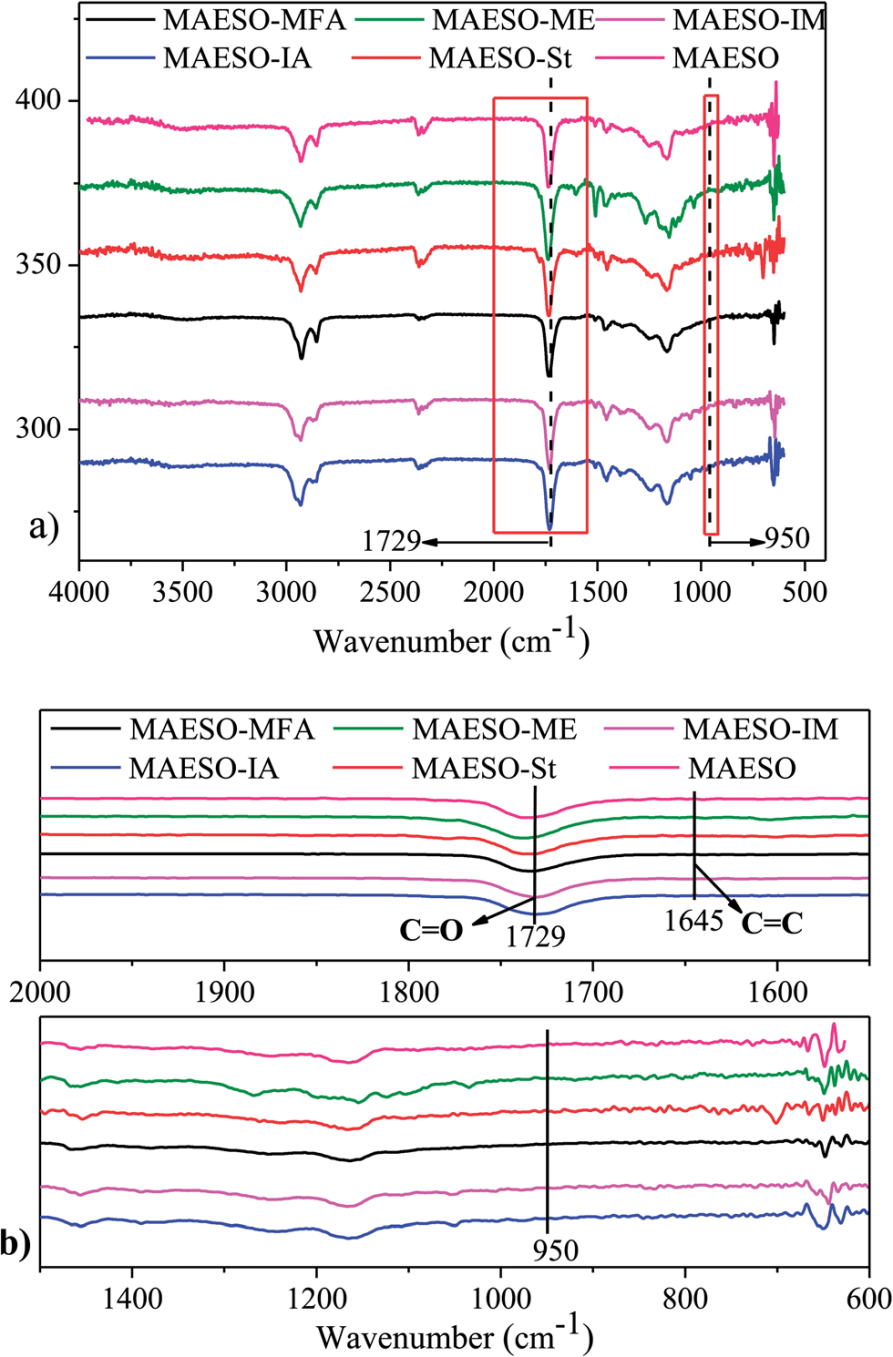
FT-IR spectra of cured MAESO-RDs resins in the range of 4000–400 cm^−1^ (a), 2000–1500 and 1500–600 cm^−1^ (b).

Soxhlet extraction was performed for 24 h using dichloromethane as a solvent to evaluate the curing extent. The insoluble content of the cured MAESO-RD resin ranged from 90.6–93.2% (Table S6†), indicating that most of our RDs monomers were tied into the crosslinking network. The extent of cure for MAESO-RD resins was further measured by FT-IR spectroscopy, as shown in Fig. 7. After polymerization, all the MAESO-RD samples showed a strong characteristic peak at 1729 cm^−1^ in Fig. 7(a), which corresponds to the stretching vibrations of the carbonyl group (–C=O–) in the meth(acrylate) groups. There were no C=C double bonds (1645 cm^−1^) and methacrylate groups (950 cm^−1^) available in the MAESO-RD resin systems in Fig. 7(b), indicating that almost all the C=C double bonds within MAESO-RD systems have been polymerized during the free radical polymerization, further confirming high double bond conversions in the MAESO-RD systems. Furthermore, there was no obvious exothermic peak detected for five cured MAESO-RD samples in the DSC test performed from room temperature to 250 °C at 10 °C min^−1^.

**Fig. 8:**
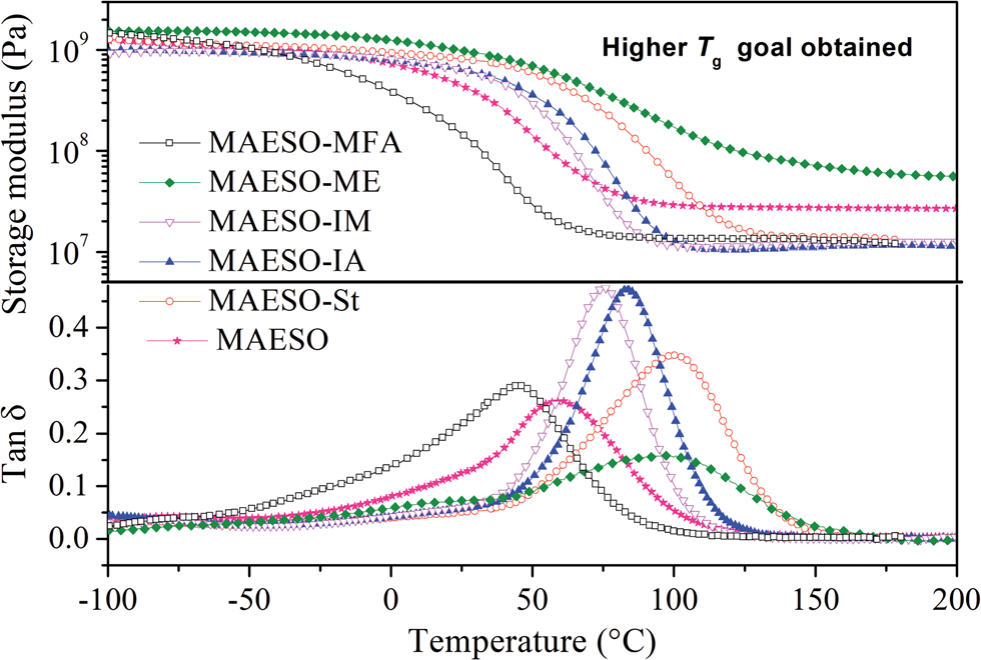
Storage modulus and tan *δ* as function of temperature for MAESO-RD resins.

The storage modulus and tan *δ* of the MAESO-RDs were obtained from dynamic mechanical analysis as shown in Fig. 8. Pure MAESO resin exhibited a storage modulus of 445.6 MPa at room temperature (25 °C). In the case of MAESO-MFA resin, the storage modulus was lower than that of pure MAESO resin. This was attributed to the introduction of the long, aliphatic chain of MFA, since long aliphatic chain are flexible, providing better mobility to the system. In contrast, MAESO-ME, MAESO-St, MAESO-IM, and MAESO-IA resin systems all showed higher storage modulus than that of pure MAESO resin because ME, St, IA, and IM all contain short and rigid structure (benzene ring and isobornyl ring).

Pure MAESO resin showed a broad tan *δ* peak from −60 to 140 °C. After the introduction of RDs, the polymerization reaction was further complicated, leading to highly heterogeneous polymer crosslinked networks.

Generally, rubbery modulus is proportional to the degree of crosslinking in thermosetting resins. It is noteworthy that MAESO-ME resin system showed a higher rubbery modulus (66.5 MPa) than that of pure MAESO resin (27.6 MPa) (Table 5), implying a higher crosslinking degree of the MAESO-ME resin. This was because ME has more reactive C=C double bonds than that of MAESO in an equivalent weight, and therefore leads to an increased crosslinking degree. However, for MAESO-St, MAESO-IM, and MAESO-IA resin systems, the crosslinking degree was lower than that of pure MAESO resin. This was due to the fact that St, IM and IA all have just one reactive group, which mainly worked as chain extenders in the polymerization, leading to a decrease in the crosslinking degree.

**Table 5:** Thermo-mechanical properties of MAESO-RD thermosets

Formulations	*T*_g_ (°C)	Glassy modulus (25 °C, MPa)	Rubbery modulus (*T*_g_ + 60, MPa)
MAESO	61.1	445.6	27.6
MAESO-MFA	44.8	152.4	13.5
MAESO-ME	98.1	984.5	66.5
MAESO-IM	75.8	625.7	11.6
MAESO-IA	83.8	634.4	11.0
MAESO-St	100.8	813.5	13.9

Generally, the intensity (height) of the tan *δ* indicates the extent of mobility of the polymer chain segments. Higher tan *δ* indicates more viscous behavior, whereas lower tan *δ* indicates more elastic behavior. The incorporation of St, IM, IA and MFA in the MAESO resin resulted in an increase in intensity of the tan *δ* peak than that of pure MAESO resin. This was because that St, IM, IA and MFA are all monofunctional RDs which worked as chain extender and cannot form crosslinking network, thereby increasing chain mobility. In contrast, ME monomer has two reactive C=C double bonds which worked as crosslinkers during the polymerization. Therefore, MAESO-ME resin system exhibited a decrease in intensity of the tan *δ* peak because crosslinking structure restricted the motion of molecular chains.

The glass transition temperature (*T*_g_) is defined as the temperature at which a maximum of tan *δ* is observed. Pure MAESO resin showed a *T*_g_ of 61.1 °C. With the introduction of MFA monomer, the MAESO-MFA resin showed a decreased *T*_g_ (44.8 °C). This was attributed to the long, flexible chains of MFA, which increased the length of the linear segments and contributed to the free volume of resulting polymer network. In the case of MAESO-IM and MAESO-IA resin, the *T*_g_ was increased to 75.8 and 83.8 °C, respectively. This was mainly attributed to the structural rigidity of the IM and IA monomer (isobornyl bis-cyclic group). The *T*_g_ of MAESO-ME system was increased to 98.1 °C, which was comparable to that of MAESO-St resin (100.8 °C). This was caused by the introduction of aromatic structure of ME. Moreover, the crosslinking degree of MAESO-ME resin was significantly higher than that of MAESO-St resin. During the polymerization of MAESO-RD system, ME worked as a crosslinker of MAESO resin, while St worked as a chain extender of MAESO resin.

**Fig. 9:**
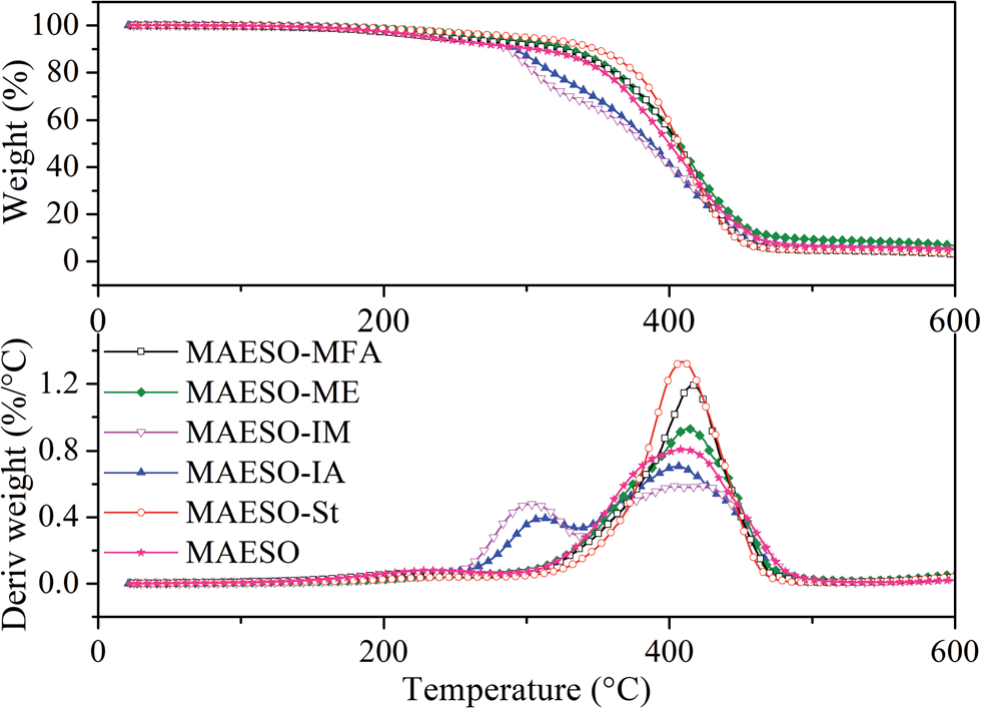
TGA and DTG curves of MAESO-RD resins.

The thermal degradation behavior of the cured MAESO-RD systems was investigated using TGA under a nitrogen atmosphere, as shown in Fig. 9. All the MAESO-RD resins were stable up to 250 °C. TGA curves of the MAESO-IM and MAESO-IA showed two stages of degradation. The first degradation (250–340 °C) showed a weight loss of approximately 30%. This was caused by the loss of isobornyl fragment by ester scission,^39^ which was in agreement with the fact that the MAESO-IM and MAESO-IA resin contained 35% of IM and IA. The second degradation (340–500 °C) was attributed to the random scission of the MAESO-IM and MAESO-IA structure. The TGA curves of MAESO, MAESO-St, MAESO-ME, and MAESO-MFA showed a single stage of degradation. The thermal stability of MAESO resin was lower than that of the MAESO-St and MAESO-ME resin. This was attributed to the introduction of more stable aromatic structure of St and ME in MAESO resin. In addition, after 410 °C, MAESO-ME resin exhibited the highest thermal stability because of the increased crosslinking degree.

## 4. Conclusion

A renewable reactive diluent ME was successfully synthesized using eugenol and methacrylic anhydride. The resulting ME and three other commercially available renewable monomers, MFA, IA, and IM were employed as RDs for MAESO resin to replace the use of styrene, as well as to produce bio-based thermosetting resins by free radical polymerization. The four RDs and MAESO resin are derived from bio-based renewable resources and have high sustainability quantified by the bio-based carbon content. Incorporation of these RDs showed great advantages in term of low VOC emissions, low toxicities, low viscosities, high sustainability, and tailored *T*_g_. ME monomer was determined to be the most promising, sustainable reactive diluent to replace styrene for MAESO resin.

## Conflicts of interest

The authors declare no competing financial interest.

## Supplementary Material

RA-008-C8RA00339D-s001
