# Supplementary material for: Bio-based reactive diluents as sustainable replacements for styrene in MAESO resin
Source: RSC Adv. 2018 Apr 12;8(25):13780–8. doi: 10.1039/c8ra00339d (PMC9079840; doi:10.1039/c8ra00339d)
Supplement: RA-008-C8RA00339D-s001 [file RA-008-C8RA00339D-s001.pdf]

Electronic Supplementary Material (ESI) for RSC Advances.
This journal is © The Royal Society of Chemistry 2018

Supporting information
For:

Bio-Based Reactive Diluents as Sustainable Replacement for Styrene in MAESO Resin

Yuehong Zhang[a,b], Yuzhan Li[b], Vijay Kumar Thakur[b,d], Liwei Wang[b], Jiyou Gu[a], Zhenhua Gao[a*],Bo Fan[a], Qiong Wu[c], and Michael R Kessler[b,e*]

[a] *College of Material Science and Engineering, Northeast Forestry University, Harbin 150040, PR China*
[b] *School of Mechanical and Materials Engineering, Washington State University, Pullman, WA 99164, USA*
[c] *College of Chemical Engineering, Qingdao University of Science and Technology, Qingdao, Shandong province 266042, PR China*
[d] *Enhanced Composites and Structures Center, School of Aerospace, Transport and Manufacturing, Cranfield University, Bedfordshire MK43 0AL, UK*
[e] *Department of Mechanical Engineering, North Dakota State University, Fargo, ND 58108, USA*

*Corresponding Author
* P.O. Box 6050, Fargo, North Dakota 58108-6050; Phone (701) 231-7494; E-mail: Michael.R.Kessler@ndsu.edu.

Total number of Pages: 7 (S1-S9)
Total number of figures: 6 (Figure S1-Figure S6)
Total number of tables: 5 (Table S1-Table S6)

List of supplementary figures and tables

Fig. S1 Schematic for the synthesis of MAESO

Fig. S2. Dynamic DSC results for MAESO at different heating rates of 5, 10, 15 and 20 °C/min

Fig. S3. Dynamic DSC results for MAESO-IA at different heating rates of 5, 10, 15 and 20 °C/min

Fig. S4. Dynamic DSC results for MAESO-MFA at different heating rates of 5, 10, 15 and 20 °C/min

Fig. S5. Dynamic DSC results for MAESO-ME at different heating rates of 5, 10, 15 and 20 °C/min

Fig. S6. Dynamic DSC results for MAESO-IM at different heating rates of 5, 10, 15 and 20 °C/min

Table S1 Peak temperature and enthalpy of MAESO resin system

Table S2 Peak temperature and enthalpy of MAESO-IA resin system

Table S3 Peak temperature and enthalpy of MAESO-MFA resin system

Table S4 Peak temperature and enthalpy of MAESO-ME resin system

Table S5 Peak temperature and enthalpy of MAESO-IM resin system

Table S6 Insoluble weight percent for MAESO-RD thermosets systems

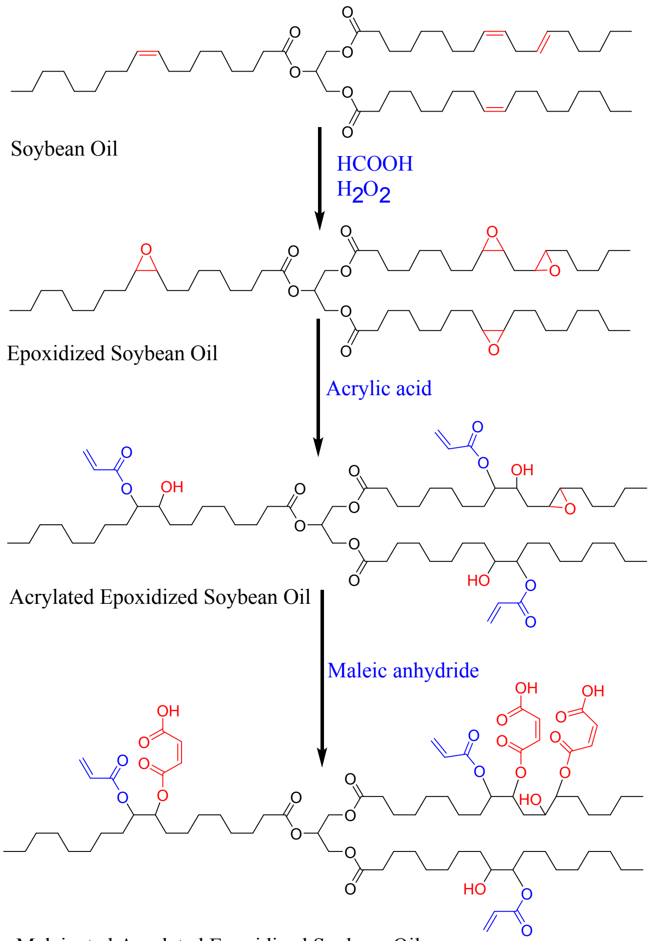

Fig. S1 Schematic for the synthesis of MAESO

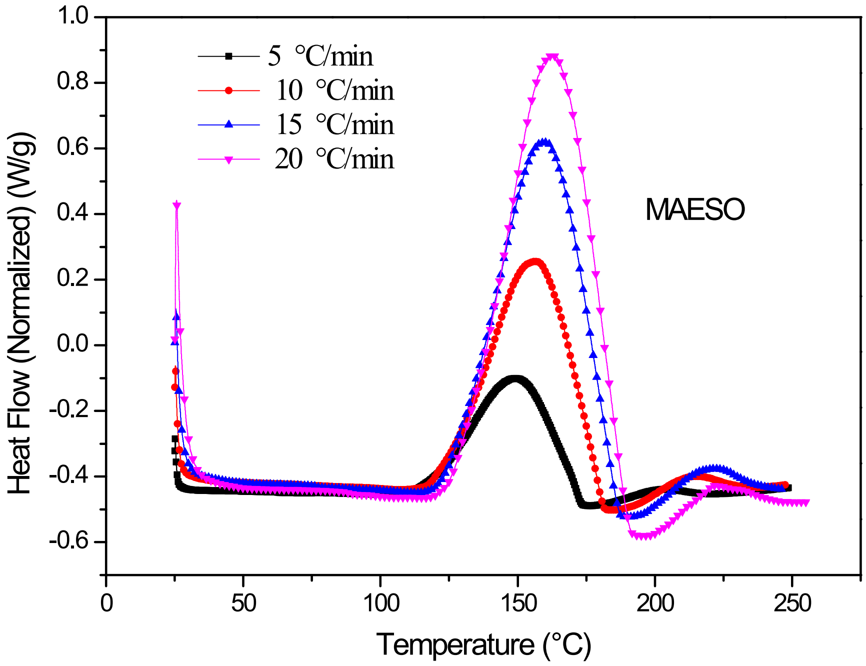

Fig. S2 Dynamic DSC results for MAESO at different heating rates of 5, 10, 15 and 20 °C/min

Table S1 Peak temperature and enthalpy of MAESO resin system

**Resin**	**Ramp (°C/min)**	**Peak temperature (°C)**	**Enthalpy (J/g)**	**Peak temperature (°C)**	**Enthalpy (J/g)**
**MAESO**	**5**	149.346	154.36	201.587	7.86
	**10**	156.965	154.28	211.506	9.56
	**15**	160.532	161.38	217.916	10.60
	**20**	162.856	158.27	221.705	8.95

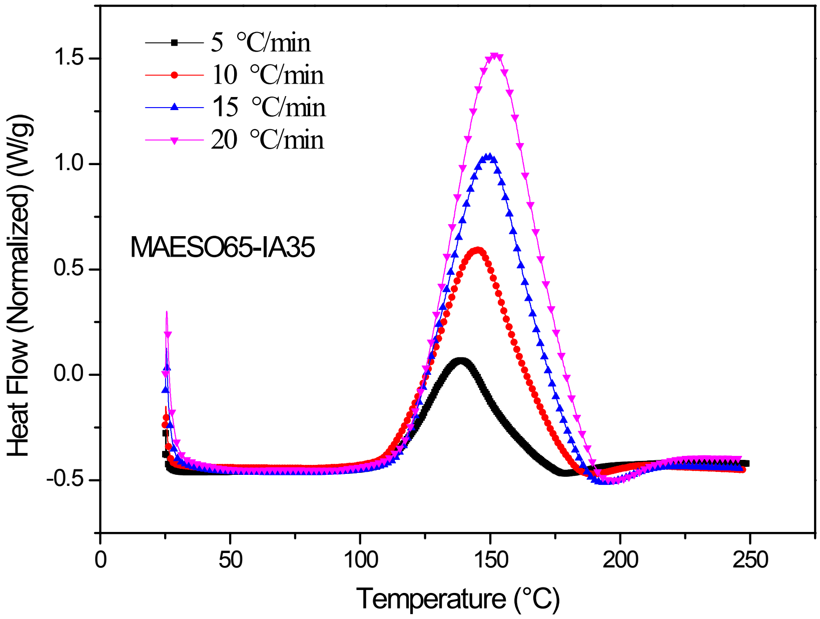

Fig. S3 Dynamic DSC results for MAESO-IA at different heating rates of 5, 10, 15 and 20 °C/min

Table S2 Peak temperature and enthalpy of MAESO-IA resin system

**Resin**	**Ramp (°C/min)**	**Peak temperature (°C)**	**Enthalpy (J/g)**
	**5**	138.657	215.34

**MAESO-IA**	**10**	145.79	234.16
	**15**	150.091	230.58
	**20**	152.236	234.17

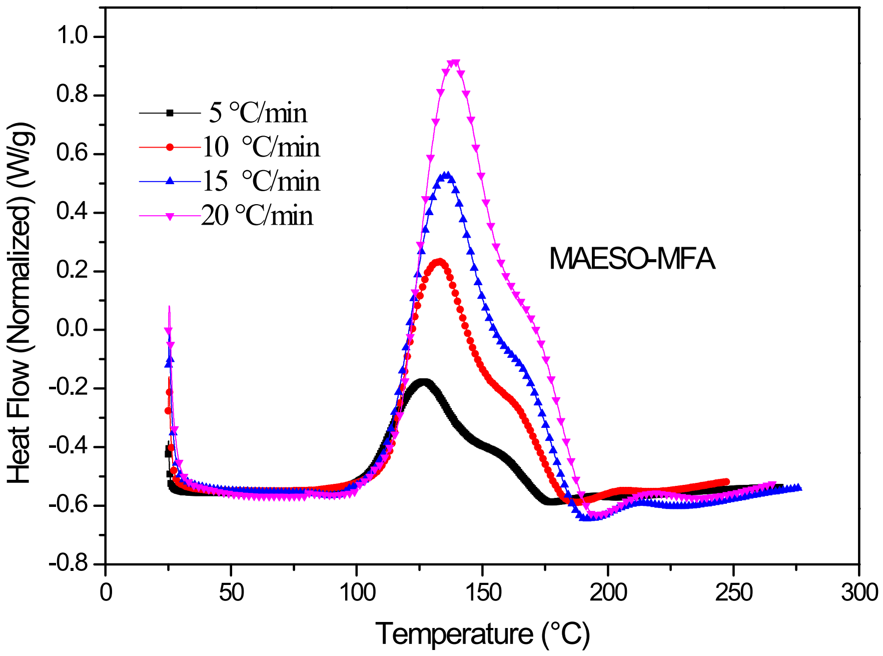

Fig. S4 Dynamic DSC results for MAESO-MFA at different heating rates of 5, 10, 15 and 20 °C/min

Table S3 Peak temperature and enthalpy of MAESO-MFA resin system

**Resin**	**Ramp (°C/min)**	**Peak temperature (°C)**	**Enthalpy (J/g)**	**Peak temperature (°C)**	**Enthalpy (J/g)**

**MAESO-MFA**	**5**	126.745	186.745	193.281	1.42
	**10**	133.285	187.25	204.862	2.03
	**15**	136.515	186.33	211.283	2.35
	**20**	139.046	187.00	215.89	2.93

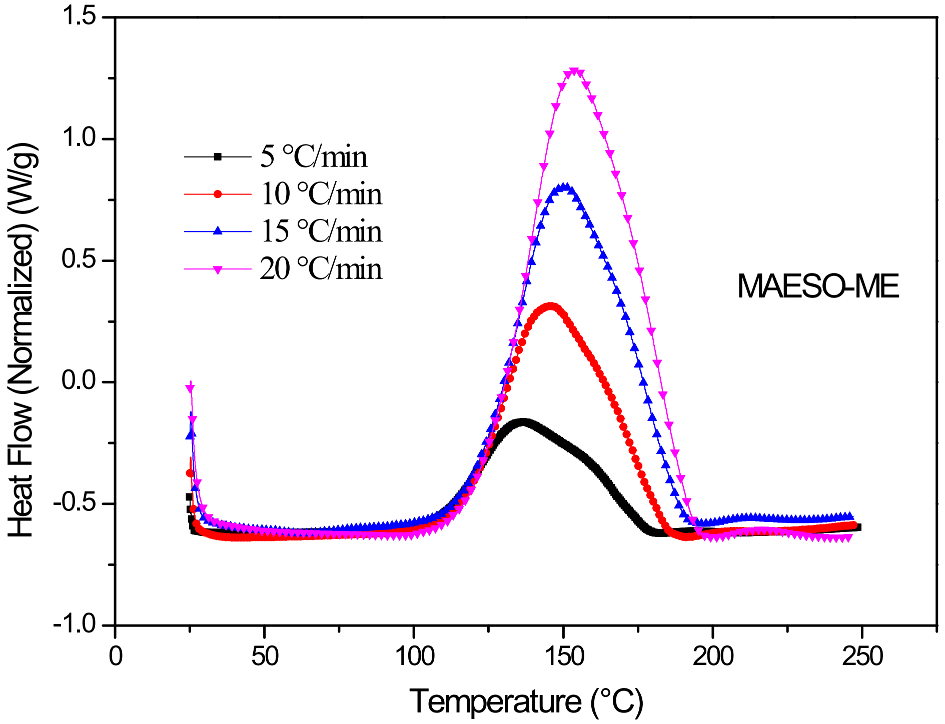

Fig. S5 Dynamic DSC results for MAESO-ME at different heating rates of 5, 10, 15 and 20 °C/min

Table S4 Peak temperature and enthalpy of MAESO-ME resin system

**Resin**	**Ramp (°C/min)**	**Peak temperature (°C)**	**Enthalpy (J/g)**

	**5**	136.699	236.69
**MAESO-ME**	**10**	145.997	238.75
	**15**	150.642	238.78
	**20**	154.225	242.59

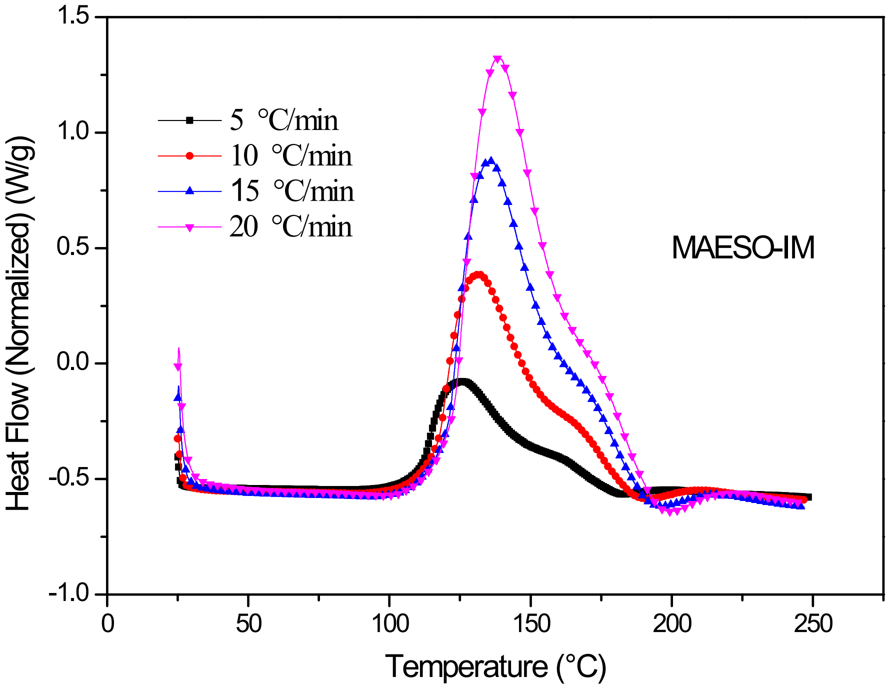

Fig. S6 Dynamic DSC results for MAESO-IM at different heating rates of 5, 10, 15 and 20 °C/min

Table S5 Peak temperature and enthalpy of MAESO resin system

**Resin**	**Ramp (°C/min)**	**Peak temperature (°C)**	**Enthalpy (J/g)**
	**5**	125.835	207.14

**MAESO-IM**	**10**	131.887	210.98
	**15**	136.004	216.04
	**20**	138.733	210.84

Soxhlet extraction tests were conducted to analyze the curing extent of the MAESO-RD thermosets. Approximately 1.000 g of MAESO-RD thermoset sample was weighted (m_1) and extracted with 250 mL of refluxing methylene chloride in a Soxhlet extractor for 24 h. The remaining insoluble fraction was dried under reduced pressure and weighed (m_2). The insoluble weight percentage was calculated as 100% × m_2/m_1.

Table S6 Insoluble weight percent for MAESO-RD thermosets systems

Formulations	Insoluble weight percent (%)
MAESO	90.6 ± 0.2
MAESO-MFA	91.0 ± 0.3
MAESO-ME	93.2± 0.2
MAESO-IM	91.8 ± 0.1
MAESO-IA	92.1 ± 0.2
MAESO-St	92.7 ± 0.3
